# Next-Generation Anticoagulants: Precision Strategies for Patient-Centered Thromboprophylaxis

**DOI:** 10.3390/jpm15100490

**Published:** 2025-10-14

**Authors:** Abdulrahman Nasiri, Manal Alshammari, Rawan Alqahtani, Omar Alshaer, Eysa Alsolamy, Hamad Alghethber, Reem Alkharras

**Affiliations:** 1 College of Medicine, Imam Mohammad Ibn Saud Islamic University (IMSIU), 11623 Riyadh, Saudi Arabia; 2 Department of Hematology, Oncology Center, King Salman Specialist Hospital, 55741 Hail, Saudi Arabia; 3 Pharmacy Department, Security Forces Hospitals Program, General Directorate of Medical Services, Ministry of Interior, 11481 Riyadh, Saudi Arabia; 4 Department of Medical Oncology and Haematology, CancerCare Manitoba, Winnipeg, MB R3E 0V9, Canada; 5 Internal Medicine Department, Security Forces Hospitals Program, General Directorate of Medical Services, Ministry of Interior, 11481 Riyadh, Saudi Arabia

**Keywords:** anticoagulation, direct oral anticoagulants, thrombosis, factor XI inhibitors, bleeding risk, personalized medicine, pharmacogenomics, novel oral anticoagulants

## Abstract

Thrombosis remains a leading preventable cause of global morbidity and mortality, with conditions like venous thromboembolism and atrial fibrillation affecting millions worldwide. Traditional anticoagulants (heparins, vitamin K antagonists) require careful monitoring due to narrow therapeutic windows. Direct oral anticoagulants (DOACs) greatly improved convenience and reduced certain hemorrhagic complications (notably intracranial hemorrhage) compared to warfarin, but bleeding, drug–drug interactions, and unmet needs in special populations persist. This review highlights emerging strategies to decouple antithrombotic efficacy from bleeding risk. Novel agents targeting factor XI or XII (small molecules, antibodies, antisense oligonucleotides) have shown in early trials robust thromboembolism prevention with low bleeding. Advances in pharmacogenomics, biomarker-guided dosing, artificial intelligence risk prediction, and digital monitoring promise to personalize therapy. We discuss optimized approaches for high-risk subgroups (cancer-associated thrombosis, extremes of body weight, renal/hepatic dysfunction, pregnancy, perioperative care, and COVID-19) with citations to current evidence. Finally, we outline critical systems-level considerations, including drug accessibility, cost-effectiveness, and educational strategies, that are necessary to realize precision anticoagulation. Our synthesis is grounded in recent peer-reviewed literature and emphasizes innovations likely to improve safety and efficacy of thromboprophylaxis.

## 1. Introduction

Venous thromboembolism (VTE) is a major preventable cause of morbidity and mortality. In the United States each year, nearly 900,000 people are affected by VTE and an estimated 60,000–100,000 die of related complications [1]. Hospitalization (medical or surgical) and cancer markedly increase VTE risk, and VTE is the third leading vascular death after myocardial infarction and stroke [2]. Atrial fibrillation (AF) is the most common sustained arrhythmia; recent data suggest ~10.5 million US adults have AF and more than 50 million globally [3]. AF confers a substantially elevated stroke and systemic embolism risk. Together, these thrombotic diseases impose enormous clinical and economic burdens worldwide.

Table 1 summarizes key epidemiologic burdens, limitations of current anticoagulants, and the rationale for developing safer, targeted thromboprophylaxis.

Standard anticoagulants, while life-saving, have important limitations. Unfractionated heparin and low-molecular-weight heparins require parenteral administration and can cause bleeding and heparin-induced thrombocytopenia. Vitamin K antagonists (e.g., warfarin) are oral but exhibit highly variable pharmacokinetics/pharmacodynamics and drug–food interactions, such as with dietary vitamin K intake, necessitating frequent monitoring to maintain the INR in a narrow therapeutic range, a process which can be complicated by inter-observer testing variability [4,5]. Consequently, two main classes (heparin and warfarin) demand careful dose adjustment and lab testing to avoid under- or over-anticoagulation. These challenges spurred the development of fixed-dose, oral agents with predictable effects.

Direct oral anticoagulants (DOACs, targeting factor Xa (FXa) or thrombin) have largely supplanted warfarin for many indications. Meta-analyses of pivotal trials show that, compared to warfarin, DOACs (dabigatran, rivaroxaban, apixaban, edoxaban) provide comparable or better stroke/systemic embolism prevention and lower rates of intracranial hemorrhage and all-cause mortality [6]. For example, a Lancet meta-analysis reported that DOACs reduced stroke, intracranial hemorrhage, and death (versus warfarin) with similar overall major bleeding (though higher gastrointestinal bleeding). Real-world studies in VTE populations also suggest DOACs have at least equivalent efficacy and improved safety compared to warfarin. Consequently, guidelines recommend DOACs over VKAs for most nonvalvular AF and VTE patients without contraindications [7].

Despite these advances, significant unmet needs remain. Major bleeding (especially gastrointestinal) still occurs with DOACs, and no current anticoagulant entirely separates antithrombotic efficacy from hemorrhage. Important patient groups such as those with cancer, severe renal/hepatic impairment, or undergoing major surgery, were underrepresented in trials, leading to many gaps in evidence. Warfarin continues to be used when DOACs are infeasible (e.g., mechanical valves, advanced CKD, cost/access barriers). Furthermore, rising utilization of DOACs has unveiled new challenges: drug–drug interactions, lack of rapid reversal agents in some settings, and difficulties in periprocedural management. Thromboprophylaxis decisions also lack personalization; risk stratification tools for bleeding versus thrombosis remain imperfect.

This review examines innovations in thromboprophylaxis aimed at safer and more individualized care.

Scope and Search Strategy:

This narrative review synthesizes recent peer-reviewed literature on next-generation anticoagulants. We conducted a targeted search of PubMed, MEDLINE, and Embase for articles published between January 2015 and September 2025, using keywords such as ‘anticoagulation’, ‘factor XI inhibitors’, ‘DOACs’, ‘personalized medicine’, and ‘thromboprophylaxis’. Priority was given to randomized controlled trials, meta-analyses, practice guidelines, and high-impact reviews to ensure transparency and rigor.

## 2. Innovations in Anticoagulant Targets and Agents

### 2.1. Factor XI and XII Pathway Inhibitors

The coagulation cascade offers targets beyond thrombin and factor Xa. Factor XI (FXI) and its activated form FXIa are intriguing because congenital FXI deficiency causes only mild bleeding yet protects against thrombosis. Inhibiting FXIa may prevent clots while sparing normal hemostasis. Early-phase trials of multiple FXI/XIa inhibitors support this concept.

### 2.2. Small-Molecule FXIa Inhibitors

*Milvexian* is an oral FXIa inhibitor that was tested in a large Phase 2 trial in knee arthroplasty patients [8]. A dose-ranging study (AXIOMATIC-TKR) showed a clear dose–response: twice-daily *milvexian* (200 mg) achieved a VTE rate of 8% vs. 21% with enoxaparin, and bleeding rates were similarly low (major or relevant bleeding 1% vs. 2%). These results suggest potent thromboprophylaxis with only minimal bleeding. *Asundexian* is an oral FXIa inhibitor evaluated in the PACIFIC-AF Phase 2 trial in patients with AF [9]. Among 753 patients, *asundexian* (50 mg daily) achieved near-complete FXIa inhibition and had significantly fewer bleeding events than standard apixaban (fewer major or clinically relevant bleeds). However, a subsequent large Phase 3 AF trial (OCEANIC-AF) of *asundexian* was terminated early due to significantly inferior efficacy compared to apixaban [10]. This underscores that demonstrating sufficient clinical efficacy for FXIa inhibitors remains challenging. Overall, oral FXIa inhibitors can markedly reduce thrombotic events with a low risk of hemorrhage, but Phase 3 confirmation is needed.

### 2.3. Monoclonal Antibodies and Antisense Agents

Long-acting biologics are also under investigation. *Abelacimab* is a monoclonal antibody binding both FXI and FXIa. In a Phase 2 trial (single IV dose postoperatively), *abelacimab* significantly reduced VTE compared to enoxaparin (VTE rates 13%, 5%, 4% with low/med/high dose vs. 22% for enoxaparin) and virtually eliminated bleeding (0–2% across *abelacimab* arms vs. 0% with enoxaparin) [11]. The highest dose (150 mg) was most effective. These findings confirm that FXI inhibition prevents postoperative VTE while causing no observable bleeding in that trial. *Osocimab* (another anti-FXI antibody) showed similar promise in the FOXTROT trial (JAMA 2020): a single IV dose post-TKA lowered VTE versus enoxaparin without excess bleeding [12]. Meanwhile, IONIS-FXIRx (an antisense oligonucleotide against FXI) reduced factor XI levels and prevented VTE after knee surgery with minimal bleeding in a NEJM study (FXI-ASO vs. enoxaparin, N Engl J Med 2015) [13]. Taken together, targeting FXI (by antibodies or RNA interference) effectively prevents thrombosis with a very favorable safety profile.

Factor XII is an upstream target that may offer similar advantages. FXII inhibitors (e.g., the antibody garadacimab/AB023) have been studied in pilot trials for thrombosis in dialysis and hereditary angioedema. Preclinical data and small studies suggest FXII inhibition could reduce clotting on extracorporeal circuits with negligible bleeding. Further development of FXIIa inhibitors is ongoing. Other novel concepts include heparan-mimetic agents (e.g., synthrodes) and thrombomodulin analogs, although none have yet reached late-stage trials.

### 2.4. Clinical Status

Several FXI inhibitors have entered Phase 3 trials: *abelacimab* and *milvexian* for stroke prevention in AF, and FXI-ASO for AF (ongoing AF trials); *milvexian* and *osocimab* for VTE prophylaxis; AB023 for stroke prevention in hemodialysis, etc. No pivotal results are published yet (as of 2025) for most of these. If Phase 3 confirms efficacy, FXI inhibition could yield the first class of anticoagulants dissociating antithrombotic effect from bleeding risk.

Table 2 outlines the emerging FXI-targeting agents under clinical development, including their mechanisms of action, delivery platforms, and clinical trial phases.

### 2.5. Precision Anticoagulation Strategies

Personalizing anticoagulation involves optimizing dose, duration, and choice of agent for each patient. Several emerging tools aim to achieve this:

**Pharmacogenomics**: Genetic variants in VKORC1 and CYP2C9 explain a large fraction of warfarin dose variability. Genotype-guided warfarin algorithms modestly improve time in therapeutic range in some trials, but widespread genotyping remains uncommon [15]. By contrast, DOACs are given in fixed doses without genotyping. However, research is exploring genetic effects on DOAC metabolism (e.g., CES1 for dabigatran). Looking forward, polygenic risk scores and targeted genotyping may help tailor anticoagulant choice or dose, especially for VKAs [16].

**Biomarkers and monitoring**: Beyond INR and APTT, novel assays (e.g., anti-Xa levels for LMWH/DOAC, thrombin generation tests) could guide therapy. Personalized coagulation profiles might identify those at high bleeding risk. For example, high plasma levels of factor VIII or d-dimer could signal thrombotic risk. In practice, routine biomarker-guided anticoagulation is not yet established, but research is active [17].

**Machine learning risk models**: Advanced algorithms are being developed to predict thrombosis and bleeding using rich clinical data. Electronic health records, imaging, and laboratory data can be mined by AI to refine risk scores. Recent work has built models to predict bleeding in cancer-associated thrombosis, incorporating cancer type, chemotherapy, and patient factors. Machine learning might integrate multiple variables (e.g., renal function, hemoglobin, concomitant drugs) to improve on simpler scores like HAS-BLED or Khorana. While most such models are not yet in routine use, they exemplify the trend toward data-driven risk stratification [18,19].

Although most advanced AI models remain investigational, simpler validated tools like the HAS-BLED and Khorana scores are widely used in clinical practice to stratify risk. The primary challenge remains the clinical implementation of more complex models, which require seamless integration with electronic health records and rigorous validation in diverse patient populations before routine adoption.

**Digital health and wearables**: Consumer devices (smartwatches, wearable ECG monitors) are detecting arrhythmias like subclinical AF in large populations. For example, the Apple Heart Study demonstrated identification of occult AF via smartwatch atrial fibrillation alerts. Trials are underway (NIH-funded) to test whether screening for silent AF and starting anticoagulation can prevent strokes. Mobile apps and web-based tools also enable patient self-management (e.g., warfarin dose calculators, adherence reminders). Such technologies will increasingly generate real-time data to tailor anticoagulant therapy (adjusting intensity based on activity, symptoms, or biometric changes) [20,21].

**Patient-reported outcomes and engagement**: Shared decision-making tools are emerging, incorporating patient preferences (e.g., weighing bleeding risk vs. stroke prevention). Surveys indicate patients value factors like need for monitoring, reversal availability, and ease of use. Incorporating patient-generated health data (bleeding diaries, quality-of-life scores) into care can help clinicians personalize anticoagulation regimens [22].

Collectively, these precision strategies promise to move anticoagulation from a “one-size-fits-most” approach to truly individualized care. Integration of genomics, biomarkers, AI predictions, and digital data could optimize who receives anticoagulation, which agent, and what dosing and duration [23]. However, most precision tools are still under development or validation, and require prospective trials to demonstrate clinical benefit.

## 3. Optimizing Therapy in Special Populations

### 3.1. Cancer-Associated Thrombosis

Patients with cancer-associated thrombosis (CAT) present a high risk of recurrent VTE and bleeding. DOACs are now established as first-line therapy for many patients with CAT. Meta-analyses of trials (e.g., Hokusai VTE Cancer, Caravaggio, ADAM VTE) show that apixaban or rivaroxaban reduce recurrent VTE compared to low-molecular-weight heparin, though bleeding (especially gastrointestinal) may be higher in certain cancers. Current guidelines suggest using DOACs (e.g., apixaban) for cancer VTE unless contraindications (e.g., GI/genitourinary tumors with high bleed risk) exist. Treatment is generally continued as long as cancer is active, given the persistently elevated recurrence risk [24,25,26]. More evidence is needed for tailoring anticoagulation by cancer subtype, drug–drug interactions (e.g., tyrosine kinase inhibitors), and optimal duration.

### 3.2. Body Weight Extremes

Obesity and low body weight pose challenges in anticoagulation. DOAC pharmacokinetics may be altered at extremes (e.g., reduced peak levels in morbid obesity, higher concentrations in low-weight patients). The 2021 ISTH guidance suggests standard doses of apixaban or rivaroxaban can be used up to a body weight of ~120 kg (BMI ~40 kg/m^2^), whereas data for patients above this range are limited [27]. Similarly, DOACs appear effective and safe in underweight patients, though evidence is less robust. A recent review of clinical data indicates apixaban may have the most favorable profile at extreme weights. For very obese patients (BMI ≫ 40), some guidelines (EHRA 2021) advise considering drug-specific levels or even warfarin if uncertainty is high [28]. Overall, growing evidence supports DOAC use in patients at the extremes, but individualized assessment is advised. Ongoing studies and registries (e.g., Mayo’s extremes of weight cohort) should provide more guidance [29].

### 3.3. Renal and Hepatic Impairment

Severe organ dysfunction complicates anticoagulation. All DOACs have some renal clearance (dabigatran ~80% renal, edoxaban ~50%, rivaroxaban ~35%, apixaban ~27%) and are contraindicated or dose-reduced in renal failure per label. Phase 3 trials excluded patients with CrCl < 25–30 mL/min, so RCT evidence in severe chronic kidney disease (CKD) or end-stage renal disease (ESRD) is sparse. Observational analyses, however, suggest DOACs (notably apixaban) may be safer than warfarin in advanced CKD. For example, a large real-world study of CKD patients with VTE found apixaban use was associated with significantly lower rates of recurrent VTE and major bleeding than warfarin. Importantly, benefits of apixaban were seen even in ESRD, without loss of efficacy. Renal function should guide DOAC dosing (e.g., apixaban 2.5 mg BID if CrCl < 30). In patients on dialysis or with CrCl < 15, some experts now favor apixaban (with close monitoring), although data are limited. Low-molecular-weight heparin (LMWH) also requires anti-Xa monitoring in severe CKD. Warfarin remains the only option in mechanical valves or cases of end-stage hepatic failure. In advanced liver disease, coagulopathy complicates INR interpretation; DOACs are generally avoided in Child–Pugh C cirrhosis due to bleeding risk. Overall, defining safe anticoagulation in severe renal/hepatic failure is an active area of research (including whether low-dose FXI inhibitors might be safe in these settings) [7,30].

### 3.4. Pregnancy

Pregnancy is a hypercoagulable state: women are ~5-fold more likely to develop VTE during pregnancy or the postpartum period. Pulmonary embolism is a leading cause of maternal mortality. However, options are limited by fetal safety. Low-molecular-weight heparin is first-line throughout gestation, as it does not cross the placenta and has well-defined dosing. Unfractionated heparin (UFH) may be used in the peripartum period because of its short half-life. Vitamin K antagonists cross the placenta and cause fetal warfarin syndrome, so they are avoided in pregnancy (though warfarin is safe during breastfeeding). In the postpartum period, a common and safe practice is to initiate warfarin while the patient is still on therapeutic LMWH, continuing both agents until the INR is stable and within the therapeutic range for at least 48 h. DOACs are typically contraindicated antenatally due to insufficient data; if used inadvertently, they are usually stopped upon pregnancy recognition. Dosing is challenging: physiological changes (volume expansion, renal clearance increase) may alter drug levels, and standard therapeutic ranges are not established for pregnancy. Importantly, pregnant patients were excluded from all anticoagulant RCTs. Key needs include improved diagnostics (e.g., pregnancy-specific D-dimer thresholds, point-of-care ultrasound), better stratification of VTE risk in pregnancy, and studies of optimal LMWH dosing and duration (especially around delivery and postpartum) [31]. Novel agents (like the FXI inhibitors) are not currently trialed in pregnancy for ethical reasons. The primary concern is fetal safety, as the effects of investigational drugs on a developing fetus are unknown, creating an unacceptable risk of harm or birth defects (teratogenicity). This caution is informed by experience with older drugs like warfarin, which is known to cross the placenta and cause fetal warfarin syndrome, and even newer agents like DOACs are contraindicated due to insufficient safety data from major clinical trials that excluded pregnant individuals [32].

### 3.5. Perioperative Management

Interrupting anticoagulation for surgery requires balancing thrombotic and bleeding risks. For warfarin-treated patients, landmark trials (e.g., BRIDGE trial) showed that “bridging” with heparin significantly increases major bleeding with little thrombotic benefit for most AF patients [33]. Thus, current practice is to avoid bridging in low-to-moderate-risk AF and resume the VKA promptly after procedure. In practice, standardized protocols (based on drug half-lives and renal function) guide when to stop and restart DOACs without bridging. For high-risk thrombotic patients (e.g., recent stroke, mechanical valves), individualized decisions are needed. Hospital protocols and integrated anticoagulation clinics can improve consistency. A major barrier is fragmented communication among specialties [34]. Embedding evidence-based interruption schemes into electronic order sets and involving pharmacists or “anticoagulation stewardship” teams can reduce errors. Nevertheless, more data are needed on the role (if any) of bridging in extremely high-risk scenarios (e.g., left atrial appendage closure, high CHA_2_DS_2_-VASc with recent TE).

### 3.6. COVID-19 and Emerging Threats

COVID-19 highlighted the complexity of thromboprophylaxis in acute critical illness. Severe SARS-CoV-2 infection induces hypercoagulability and microvascular thromboses. Multiple RCTs and guidelines have addressed optimal anticoagulation in hospitalized COVID-19, generally endorsing prophylactic dosing unless higher doses are clinically justified. More recently, “long COVID” has been associated with persistent endothelial and coagulation abnormalities, but consensus on post-discharge anticoagulation is lacking. This uncertainty is largely driven by evidence of prolonged endothelial dysfunction and persistent hypercoagulability in some individuals with long COVID. Key research gaps include identifying which patients benefit from extended prophylaxis and determining the optimal biomarkers and duration of therapy [35]. Beyond COVID, threats like new viral pandemics or biothreats could present unique coagulopathic syndromes requiring flexible thromboprophylaxis strategies. Preparedness includes rapid guideline updates and integrated care pathways that can adapt evidence to new contexts [36].

## 4. Implementation and System-Level Considerations

### 4.1. Access, Cost, and Policy

Even the best innovations must be affordable and accessible. Although DOACs are recommended as first-line in guidelines, their high cost and uneven insurance coverage have limited use in many regions. This challenge is particularly acute in low- and middle-income countries (LMICs), where multifaceted barriers include high out-of-pocket costs, a lack of generic DOAC availability, and fragmented healthcare funding. Policy efforts aimed at centralized price negotiation and supporting local generic manufacturing are critical to improving global equity. Globally, DOACs were added to the WHO essential medicines list (dabigatran, with approval that apixaban/rivaroxaban are equivalent) only in 2019 [37]. As of 2017, only 14 of 137 surveyed countries had included a DOAC on their national essential medicines lists. Price negotiation, generics, and public funding are needed to expand global access. Health economic analyses (from JAMA, Clin Ther, etc.) generally find DOACs cost-effective versus warfarin in stroke prevention when monitoring infrastructure or adverse-event costs are high. However, local budget impact matters; policy-makers must weigh investment in broader DOAC coverage against alternative healthcare needs. In underserved populations, warfarin’s monitoring burden can be prohibitive, paradoxically making an inexpensive drug harder to use than a costly DOAC. Creative models (e.g., telemedicine INR monitoring, patient self-testing) may help mitigate such disparities.

### 4.2. Guidelines and Education

Guideline bodies (ACC/AHA, ASH, ISTH) have rapidly incorporated new evidence. For example, ASH and ESC guidelines now recommend DOACs over VKAs for most AF and VTE patients. International guidelines (e.g., for cancer VTE, COVID-19 prophylaxis) are continuously updated. Yet real-world practice often lags guidelines. Educational interventions (specialist and primary care) and decision support tools embedded in electronic health records are key to closing the gap. Furthermore, clinical trials are increasingly international and include diverse populations; broadening trial enrollment will inform guideline development for populations previously underrepresented (e.g., elderly Asians, African-Americans, global south) [38].

### 4.3. Real-World Data and Learning Health Systems

Observational studies and registries (e.g., GARFIELD-AF, XALIA, ORBIT-AF) have complemented RCT evidence. For instance, real-world AF cohorts confirm that DOAC-associated intracranial hemorrhage is far less frequent than with warfarin, consistent with trials. Such “real-world evidence” helps identify rare adverse events, adherence patterns, and outcomes in subgroups (e.g., extremes of age). Ultimately, embedding randomized trials within clinical practice (pragmatic trials) and using electronic medical records to continuously learn from patient outcomes will refine best practices. Implementation science approaches (identifying barriers, facilitators, and workflow issues) are needed to ensure that innovations translate into better care.

## 5. Limitations

Several limitations within the current body of evidence warrant consideration. The potential for publication bias, in which studies with statistically significant findings are more likely to be published, cannot be entirely dismissed. Furthermore, significant heterogeneity across the included studies regarding interventions and outcome measures may restrict the generalizability of these conclusions.

## 6. Conclusions

Thromboprophylaxis is entering a new era, with next-generation agents targeting factor XI/XII offering the prospect of safer anticoagulation, while precision tools like AI promise to personalize therapy. Although this narrative review highlights these advances, its non-systematic methodology and potential for selection bias are key limitations. Future progress will depend on large-scale, pragmatic RCTs that integrate biomarkers, AI, and patient-centered outcomes. Ultimately, integrating these scientific breakthroughs with thoughtful clinical practice and policy efforts is essential to transform thromboprophylaxis into a truly personalized therapy that maximizes benefit for each patient.

## Figures and Tables

**Table 1:** Burden of Thrombotic Disease and Unmet Needs. Major conditions (venous thromboembolism [VTE] and atrial fibrillation [AF]) impose large burdens. Each row lists estimated epidemiology, key clinical challenges, and areas requiring innovation.

Condition	Epidemiology/Burden	Key Unmet Needs	Rationale for Innovation
**Venous thromboembolism**	~900,000 cases/yr in US; 60,000–100,000 VTE deaths/yr [1]	High rates of recurrence and post-thrombotic complications; bleeding risk with anticoagulants	Safer prophylaxis (e.g., FXI inhibitors); personalized risk scoring; improved adherence to prophylactic measures
**Atrial fibrillation**	~10.5 million US adults (2024); ~52 million worldwide (GBD 2021) [3]	Stroke risk despite therapy; bleeding risk; undiagnosed cases	Safer stroke prevention (new targets, risk-based dosing); early detection via screening (wearables/EHR)
**Cancer-associated VTE**	~20% of VTE cases linked to cancer	Balancing thrombosis vs. bleeding (esp. GI cancers); limited trial evidence for many regimens	Novel agents (e.g., DOACs optimized for cancer); precision prophylaxis; biomarkers of thrombosis risk
**Other (pregnancy, etc.)**	Pregnancy increases VTE risk ~5-fold; VTE common after orthopedic surgery	Safe anticoagulation in pregnancy and postpartum; perioperative management; new hypercoagulable states (e.g., COVID-19)	Tailored strategies (e.g., drug timing protocols, multidisciplinary teams); improved diagnostics

FXI :Factor XI; GBD: Global Burden of Disease; EHR: Electronic Health Record; GI: Gastrointestinal; DOAC: Direct Oral Anticoagulant; COVID-d19: Coronavirus Disease 2019; ~: “approximately” or “about”.

**Table 2:** Next-Generation Anticoagulant Agents and Targets. Representative emerging therapies beyond current DOACs, with mechanism and clinical development status.

Agent/Class	Target/Mechanism	Clinical Status (Phase)
**FXIa small molecules**	*Asundexian*, *Milvexian*—oral FXIa inhibitors	*Asundexian*: Phase 3 (AF prevention trial); *Milvexian*: Phase 3 (AF, VTE)
**Anti-FXI antibodies**	*Abelacimab*, *Osocimab*—IV monoclonal anti-FXIa/FXI	*Abelacimab*: Phase 3 (PAION-1 AF/stroke and VTE trials); *Osocimab*: Phase 3 (arthroplasty)
**FXI antisense oligo**	IONIS-FXIRx—reduces FXI synthesis	Phase 2/3 (trials in AF and VTE prevention)
**FXII inhibitors**	Garadacimab (AB023)—anti-FXIIa antibody	Early trials (dialysis shunt clot prevention, hereditary angioedema)
**Heparin mimetics/aptamers**	Novel peptides/aptamers targeting thrombin, FXa, etc.	Preclinical/early clinical
**Multi-target agents**	e.g., combined anti-inflammatory + anticoagulant	Investigational
**Reversal agents**	Broad-spectrum (ciraparantag) for all anticoagulants [14]	Phase 2 (universal antidote)

FXIa: Activated Factor XI; AF: Atrial Fibrillation; VTE: Venous Thromboembolism; FXI: Factor XI; PAION: Pharmaceutical company developing small-molecule anticoagulants (e.g., *asundexian*); IONIS: Ionis Pharmaceuticals (developer of antisense oligonucleotide therapies such as IONIS-FXIRx) . FXIRx: Antisense oligonucleotide inhibitor of Factor XI synthesis; FXII: Factor XII; AB023: Monoclonal antibody targeting activated Factor XI (FXIa); FXIIa: ctivated Factor XII; FXa: Activated Factor X.

## Data Availability

No new data were created or analyzed in this study. Data sharing is not applicable to this article.
